# mRNA expression profiling of the cancellous bone in patients with idiopathic osteonecrosis of the femoral head by whole-transcriptome sequencing

**DOI:** 10.1097/MD.0000000000030213

**Published:** 2022-09-02

**Authors:** Da Song, Cheng-Zhi Ha, Qi Xu, Yan-Hui Hu

**Affiliations:** a Department of Orthopedics, Liaocheng People’s Hospital, Liaocheng, Shandong 252000, P.R. China.

**Keywords:** femoral neck fracture, idiopathic osteonecrosis of the femoral head, whole-transcriptome sequencing

## Abstract

Idiopathic osteonecrosis of the femoral head (INFH) seriously affects patients’ activities and is a heavy burden to society and patients’ families. Therefore, the early diagnosis and treatment of INFH is essential in reducing pain and burden. In the present study, the cancellous bone under the cartilage of the femoral head was isolated from patients with INFH and femoral neck fracture (FNF). Histological examination revealed that the bone trabecular and the medullary cavity in the INFH group compared with those in the FNF group. Whole-transcriptome sequencing (WTS), a recently applied technology, plays a significant role in the screening of risk factors associated with the onset of femoral head necrosis. Herein, WTS was used to obtain the mRNA expression profile in the cancellous bone of the femoral head isolated from 5 patients with INFH and 5 patients with FNF. Compared with the FNF group, a total of 155 differentially expressed genes were identified in the INFH group. Among these genes, 96 and 59 were upregulated and downregulated, respectively. Reverse transcription-quantitative PCR and western blot analyses revealed that leucine-rich repeat-containing 17 (LRRC17) displayed the most significantly decreased mRNA and protein expression levels between the INFH and FNF groups. The expression profile of the differentially expressed genes and LRRC17 protein in the INFH and FNF groups was consistent with that obtained by WTS. LRRC17, a leucine repeat sequence, plays a significant role in regulating bone metabolism, thus indicating that LRRC17 downregulation could affect bone metabolism and could be considered a key factor in the pathogenesis of INFH.

## 1. Introduction

It is widely accepted that idiopathic osteonecrosis of the femoral head (INFH) is caused by impaired blood supply to the femoral head. However, its pathogenesis remains unknown. It has been reported that several factors, such as the occurrence of femoral head necrosis, the application of glucocorticoids, long-term heavy drinking, immunity, abnormalities in the blood clotting mechanisms, and genetic factors may lead to avascular necrosis of the femoral head, characterized by femoral head collapse, structural abnormalities, and joint dysfunction.^[1]^ In China, approximately 8.12 million adult patients with INFH require surgery. However, patients undergoing total hip replacement, due to the effects of INFH on normal life, account for approximately 30% of all patients with femoral head necrosis. This is a substantial challenge for surgeons and it also brings a heavy economic burden to family and society.^[2]^

INFH is a relatively common disease worldwide, with an increasing number of new patients every year. In 2004, the number of INFH cases in Japan was approximately 11,400.^[3]^ In the United States, it is estimated that approximately 10,000–20,000 patients develop INFH each year.^[4]^ Due to the increasing incidence of INFH, studying its pathogenesis for the development of effective and novel therapeutic approaches is of significant importance.

However, to date, there are no effective methods for the early diagnosis and prevention of INFH. Although several pathological changes can be observed in the cancellous bone of patients with INFH, the number of reports on the mRNA expression profile of the cancellous bone using whole-transcriptome sequencing (WTS) is limited. Currently, WTS is a powerful tool for biological and clinical genomic studies,^[5]^ due to its wider detection range, higher genome coverage, and its increased sensitivity to detect the expression of genes with low expression levels. Compared with other probe-based methods, WTS provides better genome coverage and is, therefore, an ideal method for identifying INFH-related differentially expressed genes. The current study aimed to reveal the changes in the gene expression profile in the cancellous femoral bone between patients with femoral neck fracture (FNF) and INFH and to further explore whether the changes in the gene expression profile could affect the pathogenesis of INFH. The results of the current study could provide a theoretical basis for investigating the pathogenesis of INFH.

## 2. Materials and methods

### 2.1. Human sample collection

The present study was approved by the Ethics Committee of the Liaocheng People’s Hospital. Each participant fully understood the study and provided written informed consent. A total of 5 patients with INFH and 5 patients with FNF were enrolled in the current study. The samples from patients with FNF and INFH were collected from the Department of Orthopedics, Liaocheng People’s Hospital between February 2017 and February 2018.

### 2.2. Inclusion criteria

The inclusion criteria were as follows: patients <80 years of age; patients with stage III or IV INFH according to the Association Research Circulation Osseous and Garden classification staging systems; patients undergoing conservative treatment for INFH for more than 6 months, which was however ineffective;); patients who voluntarily donated bone tissue and signed an informed consent form.

### 2.3. Exclusion criteria

The exclusion criteria were as follows: patients who underwent surgical hip treatment; patients with a fractured hip; patients with a history of alcohol consumption, long-term treatment with high doses of glucocorticoids or with sickle cell or diving disease; patients suffering from cancer, metabolic bone, infectious, cardiovascular, rheumatoid, rheumatism or immune system diseases; patients with tobacco or alcohol abuse. Following operation, the bone tissue samples were removed and immediately frozen in liquid nitrogen. The tissue samples from all patients were fixed with paraformaldehyde and were then decalcified, embedded in paraffin, sectioned, and stained with hematoxylin and eosin (H&E) according to the standard protocol. Histopathological examinations were performed for all samples.

### 2.4. RNA extraction and detection

The samples were crushed in liquid nitrogen and total RNA was extracted from the bone tissues using a bone tissue extraction kit (Thermo Fisher Scientific, Inc.) according to the manufacturer’s instructions. To assess RNA degradation, purity, and DNA contamination, samples were run on a 1% agarose gel. The purity and concentration of the RNA samples were determined using the NanoPhotometer spectrophotometer (Implen GmbH) and Qubit2.0 Flurometer (Thermo Fisher Scientific, Inc.), respectively. RNA integrity was accurately assessed using the Agilent 2100 bioanalyzer (Agilent Technologies, Inc.).

### 2.5. Library construction and quality inspection

The library was constructed using ≥1 µg total RNA with the NEBNext^®^ UltraTM RNA Library Prep Kit (Illumina, Inc). Oligo (dT) magnetic beads were used to enrich mRNA with polyA tails and the mRNA diluted in NEB Fragmentation Buffer was randomly interrupted with divalent cations. Using random oligonucleotides as primers and fragmented mRNA as a template, the first cDNA strand was synthesized using an M-MuLV reverse transcriptase system. RNase H was used to remove the contaminated and degraded RNA. In the DNA polymerase I system, dNTPs were used as raw materials to synthesize the second strand of cDNA. The double-stranded cDNA was repaired and it was then amplified by PCR. Subsequently, the PCR products were purified to obtain the library. For quality inspection, after the library was constructed, RNA was diluted at a density of 1.5 ng/µL and the insert size of the library was detected. Reverse transcription-quantitative PCR (RT-qPCR) was performed for quantification.

### 2.6. Illumina sequencing

After the quality score of the library reached a standard value, Illumina sequencing was conducted. Illumina sequencing is based on sequencing by synthesis technology. dNTPs labeled with 4 types of fluorescent probes, DNA polymerase, and adapter primers were used for amplification. While the sequencing cluster extends the complementary strands, the added fluorescence-labeled dNTPs release the corresponding fluorescence. After the sequencer captured the fluorescent signal, a specific software was used to convert the light signal into a sequencing peak and the sequence information of a certain fragment was detected.

### 2.7. Data quality control

To ensure reliability and stability, the original data were filtered as necessary. During filtering, sequences without 3′ linkers and inserts, with 5′ linkers, reads that confirm base information, high polyAT sequences, low-quality reads, and sequences whose length was out of range, were removed. Therefore, clean data were obtained. Since stringent steps were adopted, all subsequent analyses were considered as high-quality analyses with clean data.

### 2.8. Sequence alignment to a reference genome

The gene model annotation file and reference genome were downregulated from the genome website (http://www.ncbi.nlm.nih.gov/guide/howto/submit-sequence-data) for sequence comparison. HISAT2, a fast and sensitive comparison program, can map the next-generation DNA and RNA sequencing reads to a single reference and human genome.^[6]^

### 2.9. Prediction of new transcripts

StringTie is a fast and efficient assembler that predicts genes.^[7]^ It uses a new network flow algorithm to assemble RNA sequences and quantifies the full-length transcripts of multiple splice variants. This alignment can be converted into a potential transcript.

### 2.10. Quantification of gene expression levels

The Fragments Per Kilobase of transcript per Million Mapped reads (FPKM) method is the most commonly used method for accurately estimating gene expression levels.^[8]^

### 2.11. Differential expression analysis

The DESeq2 software was used to perform the differential expression analysis between 2 combinations (2 biological replicates for each group).^[9]^ This software uses the likelihood ratio and Fisher’s exact tests. MA plot, a novel statistical method, was used to detect genes with differences in their gene expression levels and to calculate the P values obtained by different statistical calculations.^[10]^ A Benjamini-Hochberg test was applied to reduce errors and control false discovery rates. *P* < .05 was considered to indicate a statistically significant difference. A |log2 fold change (FC)| of >0 and p.adj<.05 were set as cutoff criteria to indicate significant differences between the screening groups.

### 2.12. Differential gene enrichment analysis

The clusterProfiler software was used to correct the gene length deviation.^[11]^ This program is used to perform the Gene Ontology (GO; http://geneontology.org/) enrichment analysis of differentially expressed genes, as well as to visualize and perform the statistical analyses of the functional maps of genes and gene clusters. GO is a comprehensive database consisting of 3 ontologies, namely biological process (BP), cell composition (CC), and molecular function (MF). According to the number of differentially expressed genes in each GO term, compared with the entire genome background, a hypergeometric test was carried out to identify the significantly enriched GO terms. After multiple test corrections, GO terms with a *P* value <.05 were considered statistically significant.

Kyoto Encyclopedia of Genes and Genomes (KEGG; http://www.kegg.jp/) is a database that includes the whole genome and all metabolic pathways.^[12]^ It is used to obtain information regarding the expression levels of specific genes. Therefore, large-scale molecular datasets are generated through genome sequencing and high-throughput databases. The data analysis provides information regarding several advanced functions in different biological systems such as cells, organisms, and ecosystems. In the present study, the cluster Profiler software was used to analyze the differentially expressed genes in the KEGG pathway and to perform statistical enrichment.

### 2.13. Verification of WTS

Bone tissue specimens were collected from 5 patients from each group. The expression levels of the first 4 genes with the most significantly increased and decreased expression levels in the WTS were verified by RT-qPCR and western blot analysis.

Total RNA was extracted using TRIzol^®^ reagent (Invitrogen; Thermo Fisher Scientific, Inc.) and the RNA concentration and purity were then determined using the NanoDrop 2000 Spectrophotometer (Invitrogen; Thermo Fisher Scientific, Inc.). Subsequently, 1 µg total RNA was reverse transcribed into cDNA using the PrimeScript™ RT reagent kit (Takara Bio, Inc.). The thermocycling conditions used for RT-PCR were: 37°C for 15 min, 85°C for 5 s, and cooling to 4°C. Subsequently, qPCR was performed on the ABI 7500 Sequence Detection System (Applied Biosystems; Thermo Fisher Scientific, Inc.) using the SYBR^®^ Premix Ex Taq™ II kit (Takara Bio, Inc.). The thermocycling conditions for qPCR were the following: Preincubation at 95 °C for 30 s for 1 cycle, followed by 40 cycles at 95°C for 5 s and 60°C for 34 s. The primers were synthesized by Sangon Biotech Co., Ltd. The primer sequences used are listed in Table 1. The housekeeping gene GAPDH served as an internal control and the 2^−ΔΔCq^ method was used to calculate the relative expression levels of each gene.^[13]^ Each experiment was independently repeated 3 times with the same conditions.

**Table 1:** Primer sequence.

Gene	Refseq Accession	Directionn	Sequence (5′ to 3′)
HLA-DQB1	NM_001243962	Forward	CCTCAACCACCACAACCTGCTG
		Reverse	AGCTGTCTCCTCCTGGTCATTCC
AJAP1	NM_018836	Forward	GGCTGTCCATCAGATCATCACCATC
		Reverse	GTGGCTGTTCCGACGAGTGTTC
MATN3	NM_002381	Forward	GCCACTGAGGAAGCACGAAGAC
		Reverse	GAGCTGACCTTGTCCTGGAATGC
SLCO2A1	NM_005630	Forward	AGCAGATGAAGCAAGGAAGTTGGAG
		Reverse	CCAGGACCACCAGGACGAAGAG
LRRC17	NM_001031692	Forward	AAAGTGCCAAACAACATCCCT
		Reverse	TGGGTCGAAGTTGGTTGATTTT
ADM	NM_001124	Forward	GGTTTCCGTCGCCCTGATGTAC
		Reverse	AGAGCCCACTTATTCCACTTCTTTCG
CHST7	NM_019886	Forward	CTCTTCATGTCAACGTTTCTGG
		Reverse	GAGCCAAAGATGACCAAGTTAC
IL1B	NM_000576	Forward	AAAGAGCGAAAGAAAGAAGTGG
		Reverse	CTTCATCTTCCTCCTCTCCATC

### 2.14. Western blot analysis

The proteins were extracted from the bone tissues of 5 patients with FNF and 5 patients with INFH using a protein extraction kit (Invent Biotechnologies, Inc.). The genes with the most significantly decreased expression levels were selected for verification. The protein concentrations were quantified using the BCA method. Total protein samples (25 μg) were separated by SDS-PAGE and immunoblotting was performed using primary antibodies against LRRC17 (1:600, PA5-113953, Thermo Fisher Scientific, Inc.). The protein expression levels of LRRC17 were normalized to those of β-actin (Thermo Fisher Scientific, Inc.). Finally, a western blot imaging system (Bio-Rad Laboratories, Inc.) was used to analyze and quantify the band intensity.

### 2.15. Statistical analysis

All data are expressed as the mean ± SD from at least 3 independent experiments. Quantitative data were recorded and statistical analyses were performed using SPSS 20.0 software (IBM Corp.). The differences between 2 groups were compared using an independent sample Student’s *t*-test. *P* < .05 was considered to indicate a statistically significant difference. GAPDH was used as the internal reference gene and the 2^−ΔΔCq^method was used to calculate the relative gene expression levels.^[14]^

## 3. Results

### 3.1. Macroscopic observation of the bone tissue in the FNF and INFH groups

**Figure 1.**
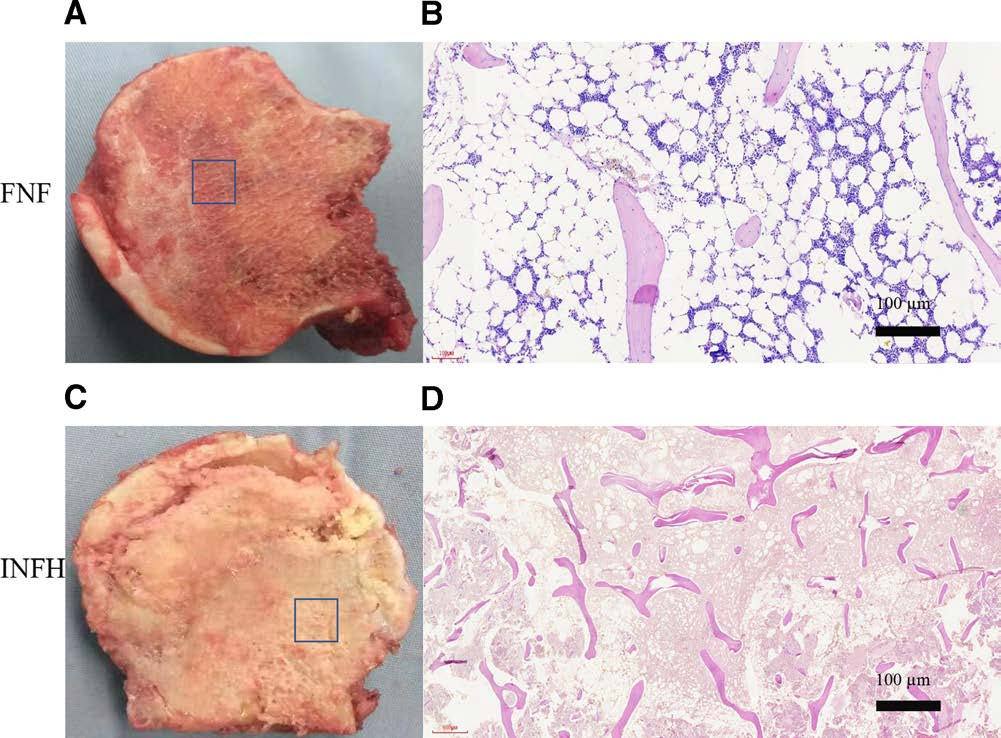
(A and B) H&E staining of the longitudinal section of the femoral head tissue in patients with femoral neck fracture (scale bar, 50 µm). (C and D) H&E staining of the longitudinal section of the femoral head tissue in patients with idiopathic osteonecrosis of the femoral head (scale bar, 50 µm). H&E, hematoxylin and eosin.

The surface of the femoral head in the FNF group was smooth and round, the cartilage was relatively complete and the color was white. Following a longitudinal incision along the coronal position, the peripheral bone tissue was yellow, while the bone tissue near the center was red (Fig. 1A). However, the surface of the femoral head in the INFH group was rough and uneven. The cartilage and subchondral bone were separated, while after longitudinal incision, the local necrotic area was small, yellow, and boneless. The beam structure was filled with yellow sand granular tissue, whereas the bone tissue was relatively loose, soft, and fragile to touch (Fig. 1C).

### 3.2. Histological analysis

The trabecular bones in the femoral head of patients with FNF were neatly and completely arranged. In addition, new bone formation and active bone metabolism were also observed (Fig. 1B). Compared with the FNF group, the trabecular bone structure in the INFH group was damaged. In addition, the bone trabecula was sparse, with an increased number of fibroblasts in the granular tissue of the medullary cavity and with varying amounts of collagen (Fig. 1D).

### 3.3. Differential expression analysis

**Figure 2.**
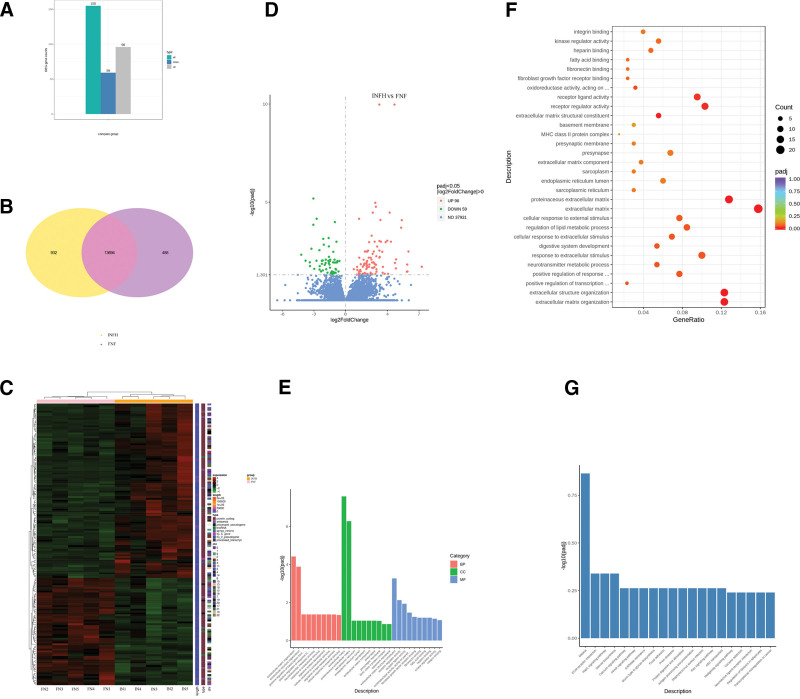
Differential expression analysis and differential gene enrichment analysis. (A) Compared with the femoral bone tissues from patients with femoral neck fracture, a total of 155 differentially expressed mRNAs were identified in the idiopathic osteonecrosis of the femoral head group, including 96 upregulated and 59 downregulated genes. (B) The circle in the Venn diagram represents the total number of differentially expressed genes obtained in the present study. The overlapping area represents the number of differentially expressed genes shared between both groups. (C) A cluster diagram of differential gene distribution is presented. The abscissa in the figure contains the name of the sample and the ordinate is the value of the differentially expressed gene. The redder the color, the higher the expression levels are. Consistently, the greener the color, the lower the expression levels are. The heatmap shows the chromosome to which each gene belongs, the length of the gene, and its biological type. (D) The abscissa in the figure indicates the fold change of gene expression in both groups, while the ordinate indicates whether the difference in the gene expression levels between the 2 groups is significant. Red and green dots indicate upregulated and downregulated genes, respectively. (E) The abscissa in the figure represents GO terms and the ordinate represents the significance level of GO term enrichment. The higher the values are, the more significant the enrichment is. Orange, green, and blue colors indicate the 3 GO functions, namely biological process, cell composition, and molecular function, respectively. (F) In the GO enrichment analysis results, the most significant terms were selected to construct a scatter plot. The abscissa represents the ratio of the number of differentially expressed genes annotated to GO terms to the total number of differentially expressed genes. The ordinates represent the GO terms. The larger the dots, the larger the increase was in the number of genes annotated to the GO term and *vice versa*. The size represents the annotation in the GO terms. The number of genes in GO terms is also shown. The change from red to purple indicates a significant change from large to small. (G) The abscissa represents the Kyoto Encyclopedia of Genomes and Genomes pathway and the ordinate indicates the level of significance of pathway enrichment. The higher the value is, the higher the significance level is. GO, Gene Ontology.

Compared with the femoral bone tissues isolated from patients with FNF, a total of 155 differentially expressed genes were detected in the INFH group, including 96 upregulated and 59 downregulated genes (Fig. 2A). Venn diagram analysis revealed 13,694 differentially expressed overlapped genes (Fig. 2B). Heatmaps (Fig. 2C) and volcano maps (Fig. 2D) were constructed to visualize the genes with significant differences. The downregulated or upregulated mRNAs in the INFH group compared with the FNF group are listed in Table 2.

**Table 2:** The 10 upregulated and 10 downregulated mRNAs with the most significant changes in expression.

Upregulated mRNA					
gene_id	log2 FoldChange		*P*adj	gene_biotype	gene_name
ENSG00000179344	4.662431424		1.06E-10	protein_coding	HLA-DQB1
ENSG00000196581	3.218055263		1.06E-10	protein_coding	AJAP1
ENSG00000132031	2.831530829		1.07E-05	protein_coding	MATN3
ENSG00000174640	2.889436166		1.72E-05	protein_coding	SLCO2A1
ENSG00000011201	2.597042211		3.40E-05	protein_coding	ANOS1
ENSG00000196735	4.20565013		3.40E-05	protein_coding	HLA-DQA1
ENSG00000213316	1.292843678		7.01E-05	protein_coding	LTC4S
ENSG00000174175	3.324633898		8.05E-05	protein_coding	SELP
ENSG00000145423	5.360671909		8.25E-05	protein_coding	SFRP2
ENSG00000070193	5.296332826		0.000199	protein_coding	FGF10

Downregulated mRNAs					
gene_id		log2 FoldChange	*P*adj	gene_biotype	gene_name
ENSG00000147872		−3.054673456	6.52E-06	protein_coding	LRRC17
ENSG00000148926		−2.741973345	7.01E-05	protein_coding	ADM
ENSG00000147119		−1.008915273	0.0001032	protein_coding	CHST7
ENSG00000125538		−3.063403039	0.0001415	protein_coding	IL1B
ENSG00000081181		−1.602345185	0.000672	protein_coding	ARG2
ENSG00000164687		−2.120008686	0.0007363	protein_coding	FABP5
ENSG00000052802		−0.859665443	0.0010321	protein_coding	MSMO1
ENSG00000234964		−2.06709908	0.0014699	_pseudogene	FABP5P7
ENSG00000139289		−1.583557668	0.0017921	protein_coding	PHLDA1
ENSG00000267583		−3.244917008	0.0023519	lincRNA	AC007998.3

### 3.4. Enrichment analysis

A histogram was constructed based on the 3 major categories, namely BP, CC, and MF, to reveal the upregulated and downregulated genes (Fig. 2E). The GO enrichment results are shown in Table 3. A scatter plot was constructed using the 30 most significantly enriched terms in the GO enrichment analysis (Fig. 2F). Furthermore, a histogram was constructed to visualize and analyze the most significantly enriched pathways in the KEGG enrichment analysis (Fig. 2G).

**Table 3:** Functional analysis results of differential mRNA.

Category	GOID	*P*adj	Description	Count
BP	GO:0030198	3.78E-05	Extracellular matrix organization	16
BP	GO:0043062	0.00013118	Extracellular structure organization	16
BP	GO:1990440	0.04233868	Positive regulation of transcription from RNA polymerase	3
			II promoter in response to endoplasmic reticulum stress	
BP	GO:0032103	0.04233868	Positive regulation of response to external stimulus	10
BP	GO:0042133	0.04233868	Neurotransmitter metabolic process	7
BP	GO:0009991	0.04233868	Response to extracellular stimulus	13
BP	GO:0055123	0.04233868	Digestive system development	7
BP	GO:0031668	0.04233868	Cellular response to extracellular stimulus	7
BP	GO:0019216	0.04322275	Regulation of lipid metabolic process	11
BP	GO:0071496	0.04602682	Cellular response to external stimulus	10
BP	GO:0021983	0.04602682	Pituitary gland development	4
BP	GO:0031667	0.04602682	Response to nutrient levels	12
BP	GO:0015732	0.04602682	Prostaglandin transport	3
BP	GO:0030949	0.04602682	Positive regulation of vascular endothelial growth factor	3
			Receptor signaling pathway	
BP	GO:0072574	0.04602682	Hepatocyte proliferation	3
BP	GO:0072575	0.04602682	Epithelial cell proliferation involved in liver morphogenesis	3
CC	GO:0031012	2.61E-08	Extracellular matrix	21
CC	GO:0005578	5.30E-07	Proteinaceous extracellular matrix	17
MF	GO:0005201	0.00053979	Extracellular matrix structural constituent	7
MF	GO:0030545	0.00756126	Receptor regulator activity	13
MF	GO:0048018	0.01174209	Receptor ligand activity	13
MF	GO:0016709	0.03399592	Oxidoreductase activity, acting on paired donors, with	
			Incorporation or reduction of molecular oxygen, NAD(P)H as	4
			One donor, and incorporation of 1 Atom of oxygen	

*P*adj is the corrected *P* value.

### 3.5. Verification of WTS results by RT-qPCR and western blot analysis

**Figure 3.**
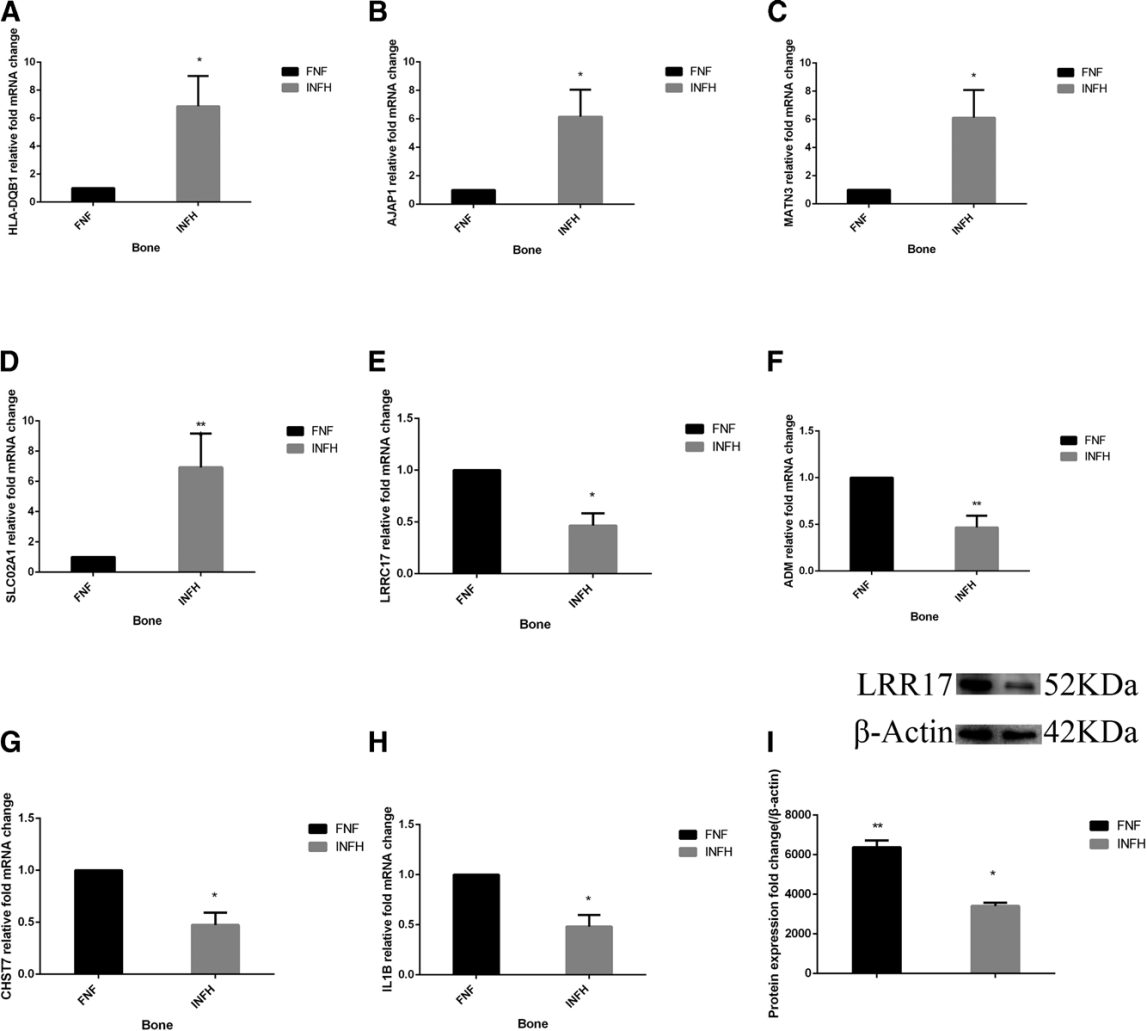
Reverse transcription-quantitative PCR and western blot results. The gene expression levels of (A) *HLA-DQB1*, (B) *AJAP1*, (C) *MATN3*, (D) *SLCO2A1*, (E) *LRRC17*, (F) *ADM*, (G) *CHST7*, and (H) *IL1B* in the femoral head tissue of patients with FNF and INFH are shown. ^*^P<0.05, ^**^P<0.01. The expression levels of all genes were normalized to those of the internal reference gene GAPDH. The relative gene expression levels were analyzed using the 2^-ΔΔCq^ method. (I) The differences in the protein expression levels of LRRC17 in bone tissues between patients with INFH and FNF are shown. β-actin served as an internal control. ^*^P < .05, ^**^P < .01. FNF, femoral neck fracture; INFH, idiopathic osteonecrosis of the femoral head.

The WTS results showed that the 4 most significantly upregulated mRNAs in the bone tissue of patients with INFH were *HLA-DQB1*, *AJAP1*, *MATN3*, and *SLCO2A1*. Consistently, the 4 most significantly downregulated mRNAs were *LRRC17*, *ADM*, *CHST7*, and *IL1B*. Additionally, RT-qPCR analysis demonstrated that the average expression levels of *HLA-DQB1* in the femoral head tissues of patients with INFH were approximately 6.85 times higher compared with the FNF group (*P* < .05; Fig. 3A). In addition, the average expression levels of *AJAP1, MATN3*, and *SLCO2A1* in the femoral head tissue of patients with INFH were approximately 6.15, 6.12, and 6.92 times higher, respectively, compared with patients in the FNF group (Fig. 3B–D; all *P* < .05). By contrast, the average expression levels of *LRRC17, ADM, CHST7*, and *IL1B* in the femoral head tissue of patients with INFH were approximately 0.46, 0.47, 0.47, and 0.50 times lower, respectively, compared with those in the FNF group (Fig. 3E–H; all *P* < .05). The protein expression levels of LRRC17 were detected in the bone tissues of 5 patients from each group. The results showed that LRRC17 was downregulated by 0.54 folds in the INFH group compared with the FNF group (Fig. 3I).

## 4. Discussion

Transcriptome studies can provide a better understanding of the developmental processes and the occurrence of several diseases in different organisms and are also considered as the basis for the study of gene structure and function.^[15]^

The protein-coding genes account for only approximately 1% of all genes in the human genome. Additionally, it has been reported that 70%–90% of the human genome is not involved in the protein-coding process.^[16]^ Although protein-coding genes and their transcripts account for only a very small part of the genome and transcriptome in eukaryotic cells, respectively, differences in the gene expression profile may cause changes in cell function, eventually leading to the onset of particular diseases.

Herein, differentially expressed genes were identified between the INFH and FNF groups using WTS. Different mRNA not only plays a key role in maintaining the balance between bone formation and bone resorption but also plays an important role in the transdifferentiation between BMSC osteogenesis and adipogenesis. However, it is not yet clear the relationship between mRNA expression and pathogenesis of INFH. Maybe, in the future, we would find it.

The functional analysis of the above differentially expressed genes revealed that these genes were mainly enriched in the processes of “growth”, “development”, “regulation of the extracellular matrix”, “regulation of cell metabolism”, and “response to extracellular stimuli”. These findings partially suggested that these mRNAs could serve a significant role in regulating cell metabolism and extracellular structure. Furthermore, pathway enrichment analysis revealed that the differentially expressed genes were mainly enriched in the “peroxisome proliferator-activated receptor (PPAR) signaling pathway”, “ras-related protein (Rap) signaling pathway”, “Ca^2+^ signaling pathway” and “Wnt signaling pathway”. The aforementioned signal transduction pathways can regulate several cellular functions.

It has been reported that the PPAR signaling pathway plays a key role in regulating osteogenic-adipogenic transdifferentiation of bone marrow mesenchymal stem cells (BMSCs). Therefore, when the PPAR signaling pathway is inhibited, the osteogenic differentiation of BMSCs is promoted. On the contrary, activation of the PPAR signaling pathway promotes the adipogenic differentiation of BMSC.^[17]^ Furthermore, Rap regulates several important signal pathways in cells, which are closely associated with significant biological functions such as cell proliferation and differentiation, cell polarity formation, and cell adhesion and movement.^[18]^

The Ca^2+^ signaling pathway is ubiquitous in all eukaryotic cells and is involved in several cellular processes. Therefore, Ca^2+^ signaling not only regulates intracellular physiological processes but also plays a crucial role in regulating long-distance signal propagation and other physiological responses. Wnt belongs to the highly conserved glycoprotein family and is rich in cysteine. This pathway is involved in the activation of several signaling pathways in an autocrine or paracrine manner and several developmental processes associated with embryo formation.^[19]^ In addition, a study showed that Wnt signaling was involved in regulating the differentiation and self-renewal of stem cells.^[20]^

Emerging evidence has suggested that the expression of several genes plays a key role in the pathology of femoral head necrosis.^[21]^ WTS revealed that LRRC17 expression was significantly downregulated in patients with INFH. Additionally, GO analysis showed that the function of LRRC17 was primarily enriched in the “regulation of lipid metabolism”. It has been also reported that LRRC17 is closely associated with bone production. Therefore, a study demonstrated that LRRC17 downregulation inhibited osteogenesis.^[22]^

LRRC17 is also a member of the LRR superfamily that directly regulates osteoclastogenesis. In addition to receptor activator of nuclear factor-κB ligand (RANKL), RANK, and osteoprotegerin, which serve a significant role in osteoclast differentiation, LRRC17 is considered as another important regulator of osteoclast development. Therefore, a study showed that LRRC17 exhibited a negative regulatory effect on RANKL-induced osteoclast differentiation.^[23]^

It was reported that the expression of LRRC17 in BMSCs was highly positively correlated with age. When the LRRC17 gene is knocked out, aging bone marrow stromal stem cells can recover their vitality, which has the effect of treating osteoporosis.^[24]^

The metabolism in the bone tissue changes dynamically. Therefore, inflammation, diet, exercise, metabolic diseases, and abnormal changes in lipid metabolism can alter the cell metabolism pathway, eventually resulting in corresponding changes in bone cells.^[25]^ For example, a study suggested that in hyperlipidemia the formation of osteoclasts could enhance the absorption of bone mass.^[26]^

In addition, the association of pre-B cell colony enhancing factor 1 (PBEF1) with obesity, together with its pro-inflammatory properties suggests that PBEF1 might be another crucial mediator that links inflammation with osteonecrosis. So, INFH may be related to multiple factors.^[27,28]^

Therefore, it was hypothesized that lipid metabolism was closely associated with osteoclasts, whereas osteoclasts and osteoblasts could work together to maintain the balance of bone metabolism in the body. Overall, these findings indicated that lipid metabolism was closely associated with bone metabolism.^[29]^

The differences in the expression of LRRC17 in human BMSCs and bone tissues from patients with IFHN could cause abnormal bone metabolism in the necrotic area of the femoral head to a certain extent, thus resulting in pain in patients with femoral head necrosis. Therefore, studying the role of LRRC17 in patients with INFH is of significant importance for uncovering the pathogenesis of INFH.

Overall, in the present study, using gene sequencing, a total of 155 genes were identified, that were differentially expressed in bone tissues between patients with INFH and FNF. These genes were enriched in associated pathways. Such differences could lead to the occurrence of INFH. Furthermore, RT-qPCR and western blot analysis demonstrated the mRNA and protein expression profiles obtained in the femoral head tissues of patients with INFH and FNF were consistent with those observed using WTS, thus indicating that the WTS results were highly accurate and credible.

## Author contributions

Da Song and Yan-Hui Hu designed and performed most of the investigation and data analysis, confirm the authenticity of all the raw data, and wrote the manuscript; Cheng-Zhi Ha and Qi Xu provided statistics assistance; Da Song and Yan-Hui Hu contributed to the interpretation of the data and analyses. All of the authors have read and approved the final manuscript.
